# Positive effect of village debt on land transfer: Evidence from county-level panel data of village finance in Zhejiang Province

**DOI:** 10.1371/journal.pone.0255072

**Published:** 2021-07-30

**Authors:** Chunhui Ye, Suwen Zheng, Edward Gu

**Affiliations:** 1 China Academy for Rural Development & School of Public Affairs, Zhejiang University, Hangzhou city, Zhejiang Province, China; 2 Institute for Advanced Study in Humanities and Social Sciences & School of Public Affairs, Zhejiang University, Hangzhou city, Zhejiang Province, China; Institute for Advanced Sustainability Studies, GERMANY

## Abstract

The purpose of this paper is to examine the impact of village debt on land transfer. Based on the county-level panel data of village finance and land transfer in 90 counties and 4 economic development zones of Zhejiang Province from 2013 to 2017, this paper carried out multivariate statistical analysis and set a fixed effect model to control the endogenous influence of region and time. It found that village debt as a pressure may encourage village committees to promote rural land transfer, and then especially promote land flows into agricultural firms; as a mechanism, the burden of village organization’s transactional debt (historical debt and administrative debt) is the key to promoting the rural land flow to agricultural enterprises; through further analysis, it is found that the village committee seeks "win-win" opportunities by intervening in land circulation to ensure the rights and interests of farmers and to obtain village benefits from them. This paper finds that this kind of push effect has the threshold, the debt will play a significant role in promoting when the debt rate is between 4.65% and 7.9%. In addition, there is regional heterogeneity in the contribution of debt, which exists only in plain, non-coastal and high-dependence areas. The results of this paper verify the view that "community mechanism and market mechanism are embedded and supported each other in acquaintance society" in the theory of community governance. In practice, it provides a realistic basis for policy makers to implement the policy of encouraging farmland circulation and properly deal with the problem of village debt.

## Introduction

The issue of land transfer is one of the key issues in China’s large-scale management and rural revitalization. In 1978, the household contract responsibility system, with the feature of “guaranteeing output to households,” initiated the reform of the rural grassroots agricultural management system in China. This reform realized the separation of land ownership and contract rights, enhanced farmers’ enthusiasm for farming, and greatly promoted agricultural growth. However, the current family-oriented decentralized small-scale operation mode sacrifices the efficiency of agricultural resource allocation and market transactions [1], which severely restricted the development of modern agriculture in our country [2,3]. Compared with specialized agricultural scale management subjects, small-scale farmers find it difficult to make full use of modern agricultural production methods, advanced agricultural production technology, and favorable agricultural policies, which hinders the improvement of agricultural production efficiency [4]. In addition, small-scale farmers tend to make excessive use of fertilizers and pesticides, which pollute the environment [5]. To change the current small-scale decentralized management mode of agricultural land and effectively improve agricultural production efficiency, the government has implemented a series of policies to promote the transfer of rural land management rights—that is, to promote the scale operation of agricultural land through land transfer, and to realize the synchronous development of national industrialization, information technology, urbanization, and agricultural modernization. The goal for the near future is to develop multiple forms of scaled operations under the premise of insisting on the separation of land ownership, contract rights, and management rights. In November 2014, the general office the State Council of the Central Committee of the Communist Party of China, announced the “Guiding Rural Land Management Rights Transfer with the Opinions of the Development of Moderate Scale Management of Agriculture”, which says the country should:

adhere to rural collective land ownership, the stability of peasant household contract rights, allowing flexibility of management of the land based on household contract management, advancing family management, collective management, cooperative management, enterprise management, and other common development management styles.

In 2020, the first document of the central government named by”Opinions of the Central Committee of Communist Party and the State Council of China on Doing a Good Job in the Key Areas of Agriculture, Rural Areas, and Farmers to Ensure the Building of a Moderately Prosperous Society in All Respects” emphasized the need to “encourage the development of diversified forms of appropriately sized operations and improve the agricultural socialized service system for small farmers.” Therefore, the study of rural land transfer to achieve scaled management has an important reference for the development and improvement of China’s rural agriculture.

Agricultural land transfer in China has developed rapidly under the central government’s attention and promotion, but there are also many problems. The overall scale of agricultural land transfer in China is large, but the transfer rate is still low. The land transfer mentioned here (rural land transfer) does not involve the transfer of construction land, but is limited to the narrow sense of the agricultural land transfer, especially refers to the transfer of agricultural land management rights through leasing. According to the statistics of the Ministry of Agriculture, as of the end of June 2017, the circulation area of farmland contracted by households in China was 497 million mu, with a transfer rate of 36.5%; 74.343 million households had transferred contracted farmland, accounting for 27.7% of the total number of households contracted by households. China’s transfer rate is still low compared with other countries in the world. Some scholars calculated that at the end of the 20th century, the transfer rate of agricultural land in Uruguay had reached 41%, while in the Philippines it was close to 51%, and in Bangladesh it was 42% [6]; the conversion rate in the USA was 43% [7]. In addition, land transfer is still in the early stages of development, and there are many shortcomings, such as non-standard transfer procedures, an immature leasing market, high transfer costs, the small scale, and a lack of transfer service agencies and supervision [8]. Therefore, it is of great practical significance to study how to improve agricultural land transfers in the region.

Land transfer has always been the focus of research and attention by scholars and policy-makers at home and abroad, and the central government has always emphasized the theme of its policies. However, there are still different views in the current studies on “whether increasing circulation area and realizing scaled operations will bring positive or negative effects to production and life.” Some scholars believe that land transfer can increase the planting income, increase the family income, and reduce the transaction cost of socialized services; the centralized transfer of land to farmers can effectively improve the efficiency of agricultural land resource allocation [9–11]; however, the livelihood of the elderly in rural areas has gradually attracted attention. After the transfer of land, their livelihood capital was reduced, and the livelihood risk of the elderly increased year by year with the concentration of land in the hands of a few people [12]. Therefore, the transfer/circulation problem remains a hot research area in academic circles. At present, the literature on land transfer focuses on the influence of micro farmers’ transfer behavior, which attracts more academic attention: at the macro level, there are institutional factors, policy factors, and structural factors; at the micro level, there are farmers’ social identity, economic characteristics, and cognition of policies and systems. The relatively neglected influencing factor was the middle level of rural governance. All farmers live in the countryside, and their choices and behaviors in social and economic life are undoubtedly affected by the governance of the villages in which they live. Of course, land transfer is an economic choice of the farmers themselves, but from the perspective of economic sociology, their economic choice is often embedded in the community structure and the situation in which they find themselves, especially in the social and economic structure of their community, that is, the village. As the economic structural factor of the community organization, the rural financial situation, and especially the debt situation may have a certain influence on the economic and social choices of farmers, this influence may be reflected in the imposition of constraints or the provision of opportunities. Therefore, when examining farmers’ behaviors in the land market, community embeddedness is worth exploring. Examining the impact of rural debt on land transfer provides a starting point from which to explore this perspective, and this paper makes a tentative exploration of this point.

Rural debt began to emerge in the early 21st century and gradually became a stopper knot in rural governance. Formation of rural debt can be divided into four stages: the first stage, from 1990 to 1993, saw an increase in the loss of village-run enterprises, which resulted in the first round of debt growth because the property rights of village-run enterprises are not clear, and the government continuously issued documents to clean up and recalibrate township enterprises in the face of production safety and environmental pollution problems. The second stage, from 1994 to 1997, saw the villages raise money from farmers, or borrow money from the society at large, which became the main reason for the increase of village-level debt due to “top-down” activities (universal nine-year compulsory education, road construction, etc.); the third stage, from 1998 to 2002, saw the proportion of agricultural taxation and rural pooling in township finance getting higher and higher, and local governments linked the remuneration of village cadres to the task of agricultural taxation and pooling, which pushed village cadres to adopt the policy of “clearing the upper and not clearing the lower,” that is, borrowing loans to make up for the shortage of farmers paying taxes and fees, thus aggravating the burden of village-level debt; at the fourth stage, agricultural tax was gradually canceled beginning in 2003, and was completely canceled on January 1, 2006. National financial resources began to carry out large-scale transfer payments to rural areas, and the “project for the countryside” has become a common phenomenon; to complete the project, the village collective raises money from the villagers or borrows money to make up the supporting funds needed for the implementation of the project, so new village-level debt generally appeared in the rural areas. Rural debt has a long history, and has a far-reaching impact on villages [13,14].

This paper used a two-step method to analyze the impact of village-level debt on rural land circulation by using county-level panel data from the Zhejiang Provincial Department of Agriculture from 2013 to 2017 and the macroeconomic data of the Zhejiang County Statistical Yearbook. The first step was a descriptive analysis of the data. It was found that the higher the village-level debt is, the larger the corresponding land circulation scale, and there is a significant difference in the area of different subjects flowing to it; then, through the establishment of a fixed effect model, this study finds that village-level debt can significantly affect the transfer and concentration of agricultural land, realize the aim of redistribution of land resources, promote the scale of land transfer, and change the structure of land transfer; what really boosted the scale management of land was village administration and historic debt, which promoted the flow of land to the firms; however, there is a threshold for the debt-driven effect, and the impact is significant when the debt ratio is between 4.65% and 7.9%; the village committee also participates in the land transfer and plays the role of middleman. In other words, the village complies with the policies and regulations and obtains investment income, which increases village investment income. Finally, the driving effect has regional heterogeneity and only exists in plain, non-coastal areas with a high agricultural GDP share.

The study enriches the research literature on the analysis of influencing factors of rural land transfer from the perspective of village-level finance for the first time, which not only provides a certain amount of factual evidence that village-level debt is not a bad thing, but also makes a useful supplement to the existing research on rural land transfer and village-level debt. More importantly, this study takes village-level debt as the entry point, introduces the theoretical perspective of community governance, and opens up the analysis framework of the market behavior of rural land transfer.

The rest of this paper is structured as follows. Section 2 organizes and reviews the literature on rural finance, land transfer, and finance on individual behavior, and provides a theoretical model linking village committee finance, which affects the structure of land flowing to different objects, to land transfer. Section 3 briefly introduces a model specification and an estimation strategy, and describes the data source. Section 4 makes a descriptive statistical analysis on samples to grasp the current situation of village debt and agricultural land circulation in Zhejiang Province. Section 5 reports and interprets the estimation results and examines the possible trade-off, making robust checks. Section 6 summarizes the findings and explains the policy implications.

## 2 Methods and theoretical model

### 2.1 Literature review

Regarding the issue of land transfer, domestic and foreign scholars mainly focus on the study of individual behaviors of farmers and the influencing factors of land transfer decision-making, while the relevant studies on village autonomous organizations such as village committees seldom focus on their financial situation and its impact. Therefore, this study is directly related to the three studies: first, the study of the determinants of land transfer; second, the research on rural finance; and third, the study on the behavior of finance on households, firms, and the government.

#### 2.1.1 Land transfer

Land transfer is an important issue that has drawn attention from academic circles of agricultural economy and rural development in China. Given the increasingly prominent contradictions between the traditional characteristics of small-scale agricultural production in China and the improvement in agricultural labor productivity, as well as the increase in farmers’ income and the increase in agricultural investment scale, agricultural land transfer has become an important way for farmers to expand their scale of operation and increase their income, which has received extensive attention from scholars [15]. In the existing analysis on the determinants of farmland transfer, it can be classified into the following six categories.

1. Property rights security. The unsafe property rights of farmland restrict the migration of rural labor force to cities and hinder farmers’ participation in the farmland transfer market. Some scholars have found that the security of property rights not only plays a positive role in the transfer, that is, the confirmation of property rights does not necessarily change the preference of farmers, so it will not significantly affect the transfer of farmland [16], or that property right confirmation strengthens the “endowment effect” of farmers on farmland, promotes the increase of land lease price, and has a certain inhibitory effect on farmers’ participation in land transfer [17,18].
2. Family endowments and peasant household characteristics. Some preliminary theoretical studies show that a higher ratio of non-agricultural employment of farmers can promote the transfer of agricultural land, and the free labor market will drive land leasing [19]. After that, through empirical research on the survey data of different regions, scholars found that off-farm employment or farmers’ choice of part-time employment itself does not necessarily lead to farmers’ participation in land transfer [20], and the relationship between them varies with the motivation of off-farm employment [21]. Determinants of land transfer include farmers’ characteristics variables, farmers’ own characteristics (gender, age, education level, and ability), and the characteristics of households (family population, workforce, property), farmers’ attitudes, rural social security cognition, cognitive, and farmers’ financial services such as cognitive policymaking [22].
3. Transaction costs. B.L. Luo extended the Coase Theorem, and combined with case analysis, found that the farmer’s endowment effect in the agricultural land transfer market has personality, survival, and emotional dependence, which greatly increases transaction costs and hinders land transfer [23].
4. Policy interventions. Existing studies have found that the granting of subsidies to the original contractors under the current subsidy policy reduces the probability of farmers’ participation in farmland circulation, and the government’s agricultural support policy of increasing agricultural income conflicts with the policy goal of promoting farmland circulation. In addition, farmers are subject to village-level control during farmland circulation, which significantly restrains farmland circulation [24].
5. Social networks. Based on the special national conditions of farmland in China, scholars have discussed in detail the role of trust, reputation, and social networks in farmland transfer from the perspective of sociology. Farmland is not a general commodity, and farmers’ dependence on land makes the functional attribute of land security closely related to the choice of objects for farmland transfer. Spatial distance, whether natural or social distance, causes farmers to choose to sign relational contracts with relatives and neighbors [25–27]. In short, rural land transfer behavior has the embeddedness of social relations.
6. Market factors. The market price of agricultural products affects the decision-making of farmers’ land transfer behavior [28]. However, these studies still have the following shortcomings: first, the research is mostly focused on the micro level, the analysis of stakeholders is not comprehensive enough, and the analysis of other land transfer participants is lacking; second, village committees, as grassroots self-governing organizations in rural China, as well as nodes of social and economic life in rural China, still have blind spots and deficiencies in quantitative analysis; third, land transfer as a top-down policy, and regarding the transfer of the main body of the utility and cost analysis, there are significant deficiencies.

#### 2.1.2 Rural finance

Another group of studies related to this study is rural finance. The research contents of this branch of literature can be divided into three categories.

1. Analysis of the current situation of rural finance, including village income expenditure and village debt. First, in the financial year, the village organization itself receives income. We include the village set-aside, the agricultural tax to add, the land contract cost (including mountains, forests, surface water), village enterprise fees, expropriate land compensation, village assets, family planning fines, and other income; village expenditure indicates the cost that the village organization must pay to maintain the normal operation of the village, which can be divided into the village organization operation expenditure, public investment expenditure, and debt repayment expenditure. The income and expenditure of village committees have obvious changes before and after the reform of agricultural tax. The total income of the village increased slightly, while the self-income ability obviously decreased; the total expenditure had a large proportion of continuous increase, while the structure of expenditure was more balanced [29]. Research on village-level debt can be divided into the following three types. First, there is the definition and current situation of village-level debt. Village-level debt refers to the village committee or village collective economic organizations in the management of public affairs, created during the process of economic development of all kinds of public debt management and business debt. It has existed since the 1980s, increased rapidly in the early 21st century, and is large, with varying levels of debt burden between different regions and village organizations [30]. Second, village-level debt has both positive and negative effects. A certain amount of debt is conducive to maintaining the normal operation and economic development of the village, especially for those projects that produce long-term economic and social benefits, but whose costs and expenditures are concentrated in the temporary period, which is indeed necessary [31]. However, when the amount of debt is too heavy or the village committee cannot repay the debt, this problem-heavy debt will cause great harm to the villagers, village cadres, township enterprises, financial system, county and township governments, and social stability [13,32]. Third, the formation of village debt can be attributed to four aspects: the imbalance of the “top-down” governance mechanism of the village, the large number of enterprises set up for the purpose of economic growth, the lack of public finance, and the agricultural tax [33].
2. Inadequacy of village financial management. The “village township fiscal tube” to realize the common practices of village-level financial supervision uses the many financial books and documents in the villages and towns dealing with specialized accounting management, recording the financial revenues and expenditures by the groups reported vouchers, after review by the public, but the lack of legal basis in the practice of the management system is not sound, but violations do not effectively restrict the village cadres, and because of new forces in village finances and the lack of a high-quality, talented team, many loopholes exist in village financial management [34,35].
3. Resolution of village financial problems. The solution to the village financial problems can be summed up in two aspects. First, the higher-level government actively guides and carefully divides up the debt responsibilities of local governments at all levels. On the premise of not reducing the debt service cost too much, the village committees that resolve the debt are encouraged by the mode of reward [36,37]. Second, an effective mechanism should be established to solve the existing debt by classification. For example, replacing external debt with internal debt to change the debt structure, or reducing debt by a nonlinear path can reduce welfare costs [38,39]. Moreover, a mechanism to eliminate the growth of debt should be established to increase the coverage of public finance in rural areas. At the same time, the village committee should strengthen the management of cadres and grassroots democracy construction, cut down on unreasonable expenses, establish accountability and village affairs disclosure systems, and reduce the growth of corruption in the village and the formation of a shadow economy [40,41]. The other solution would be the perfection of the market mechanism, which could reduce the credit risk of the system, improve the credibility of regional borrowers, and increase the circulation of market loans [42].

At present, there are still the following shortcomings in the study of village finance: first, the content of the study remains in the formation of fixed debt, with less analysis of its actual impact, if it is mentioned at all, and more focus on negative effects; second, due to the lack of quantitative data, research remains in the descriptive or case analysis mode.

#### 2.1.3 Individuals’ financial situation

The third group of studies focuses on the impact of an individual’s financial situation on his or her economic behavior—but this affects not only families, but also companies and governments. For households, the heavier the financial burden (the greater the debt pressure), the more significantly there will be a decrease in household expenditure [43]; at the same time, the debt repayment pressure will become a new driving force to improve productivity and profitability, which can be regarded as a major channel of rural capitalist reproduction [44]. For companies, short-term debt can help them make optimal use of leverage, restrain, and supervise the incentive effect of reducing excessive investments in low-growth enterprises, and thus help enterprises make better investment decisions [45]. For the government, a large number of theoretical studies believe that government debt will lead to higher private borrowing costs and greater uncertainty or expectation, and have a negative impact on economic growth [46,47]; however, G. Mathieu took 28 manufacturing industries in 39 developing countries and developed countries as samples and found that government debt could promote economic growth by increasing the supply of current assets or collateral through research on the difference-in-differences (DID) model [48]. Therefore, debt repayment is regarded as a channel for financing and reproduction.

Overall, both rely on theoretical framework mainly based on new institutional economics, especially in the building of new institutional economics and transaction cost economics, from property right economics to property uncertainty, and transaction costs as the foundation upon which to analyze the influence of farmers on the circulation of farmland, which combines the perspectives of behavioral economics, economics, and public economics structures, deals with the population of farmers’ social economic status and the cognitive characteristics of bounded rationality, farmers’ individual characteristics, farmers’ family characteristics, farmers’ perception of life, the cognition of rural social security, the cognition of rural land policy-making, the cognition of farmers’ financial services, and the influence of government intervention on these factors. However, the current research ignores the fact that if circulation occurs in rural areas, it is bound to be under the jurisdiction and influence of village organizations, and village committee play an important role. However, both the current theoretical analysis model and the empirical analysis framework ignore the elements of joining village committees (rural grassroots community self-governing organizations). In particular, there is no targeted analysis of the impact of village collectives on the development of rural land transfer markets from the perspective of village-level finance or village-level debt, especially the lack of empirical and empirical analysis based on the support of field data. However, most of the research on village-level debt is still in the descriptive analysis and case analysis phase, and it is seldom applied in empirical studies.

In the existing domestic and foreign literature on factors influencing land transfer, there is a lack of village debt as an influencing factor. At the same time, the impact of non-governmental organization (NGO) debt on land transfer has seldom been analyzed in the literature. Therefore, this paper uses county-level panel data of village debt and land transfer from the Zhejiang Provincial Department of Agriculture to analyze the impact of village debt on the land market from the perspective of village committees’ intervention in the land transfer market, and tests whether the mechanism of village committees’ intervention in the land transfer, which supplements existing research on the financial impact of autonomous organizations, has important academic significance; at the same time, it provides a new idea for policy-makers to resolve the “invisible” village debt and realize the “elimination of weak villages.”

### 2.2 Theoretical analysis and hypothesis

#### 2.2.1 The role of village committee in land circulation

As a grassroots social governance organization, the village committee is likely to play a role, perhaps even an important one. According to the existing literature, village committees, as intermediaries, play three different roles in the transfer of agricultural land. First, village committees, as service providers, not only take on organization and coordination, but also exercise the functions of management and supervision. The village committee provides information on the inflow and outflow, provides the negotiation mechanism and a signing place for the circulation according to the actual situation, and manages the transaction [49,50]. Second, the village committee is a rational interest subject, that is, a group of people with self-interests that serve as a rational broker organization [51]. As rational actors, the motivation of the village committee to promote the transfer of farmland is based on the premise of the collective profit of the village, and even the behavior of grabbing “gray income” furthers the interests of farmers [52]. Third, the village committee bidirectional agent role not only makes the village committee village farmland change the financial situation of most of the farmers, it helps reduce the decision-making costs, increase its price negotiation ability, work with large agent enterprises, cooperate, and promote the main body of the land, thus accelerating the speed of the village of farmland circulation [53,54].

The discussion of “acquaintance society” in Chinese literature on rural sociology appears in the discussion of community governance in the international literature. Regardless of political economy or economic sociology, community governance has become the third major governance mechanism, alongside administrative governance and market governance. Bureaucratic governance is based on command and control and mainly occurs in a hierarchical system. In the context of Williamson, it is also called “hierarchical governance.” Market governance is a voluntary transaction between non-individual market entities, based on choice and competition. Community governance, based on commitment and compliance, occurs among closely related actors who coordinate their activities based on their commitment and compliance with certain common values and norms [55–57].

From the perspective of community governance, it is natural that the behavior of the land market, especially the behavior of rural farmland transfer in China, is embedded in the rural social network and is affected by the rural governance situation. In fact, neo-institutionalists, neo-evolutionists, and neo-economic sociologists do not believe that the market, administrative, and community mechanisms are governance mechanisms that can perfectly replace each other. The relationships among the three may have both mutual inhibition and complementary embeddedness. Because most of the contracts in which the market mechanism operates are incomplete, the community and market mechanisms are embedded and they support each other, which is actually a normal condition in social and economic life, rather than a manifestation of an underdeveloped market mechanism. At the same time, the maintenance of norms, the credibility of promises, and the implementation of supervision in the community mechanism, just like the implementation of contracts in the market mechanism, all need administrative power to play a role. In the countryside, the village committee must play an active role in rural social and economic life, no doubt in many ways. In the economic life of rural society, farmland circulation is obviously not a trivial matter.

As mentioned above, it is common for the village committee to intervene in transfer as an intermediary, an independent interest subject, or a two-way agent. The extent and depth of the involvement of village committees are likely to be influenced by the state of rural economic governance, one of which is the financial situation, including debt. When faced with the pressure of debt, it is possible for village committees to promote the rise of large agricultural producers or large-scale agricultural enterprises by promoting the transfer of agricultural land.

#### 2.2.2 Theoretical model of village committees’ intervention in land transfer

As a grassroots autonomous organization, village committees intervene in the land market to gain profits not only for their own sake, but for the purpose of maximizing rural social welfare based on the village unit and achieving this goal by providing public goods, y. Therefore, this paper proposes a theoretical model of the village committee’s intervention in land circulation.

The basic goal of economic behavior is to obtain income, so the following assumptions are made in this model: first, the village committee can obtain certain income by promoting land circulation; second, the promotion of land transfer by the village committee is also accompanied by certain policy costs and transaction costs, such as policy risks and negotiation risks. The cost of the policy risk is fixed, whereas the transaction cost caused by the negotiation risk depends on the internal characteristics of the village committee. x means the transfer of land area promoted by the village committee, x>0x>0; f(x) means revenue that the village committee receives from intervention, f(x)′>0>0; C means cost of policy risk that is not influenced by x; the transaction cost of negotiation risk is M(n); n means that the internal characteristics of the village committee are not affected by x. Denote w(y) as a rural social welfare function, and w>0′>0, and let Z be a dummy variable indicating whether the village committee promotes land transfer, Z∈{0,1}∈{0,1}, 1 means push, and 0 means vice versa; let D be the financial budget of the village committee, the higher the debt of the village, the less the financial budget. The village committee then faces two interrelated choices:

1. When the village committee chooses to promote the transfer of land (i.e., Z = 1), it will bring some benefits f(x)f(x), as well as cost C and pay M(n).
2. When the village committee chooses to promote the transfer of land (i.e., Z = 1Z = 1), the village committee promotes the following:

If the village committee promotes land transfer, the village committee needs to provide public services for the village under the existing budget and maximize the social welfare of the village. The objective function is:

$$\underset{x}{\max}\;w(y)$$ (1)

s.t.y=D+f(x)−C−M(n) (2)

Whether the village committee chooses to promote the transfer of farmland depends on the social welfare that this measure will bring.

If the village committee does not promote land transfer (Z = 0), the maximum social welfare function of the village is w(D); if the village committee decides to promote land transfer (Z = 1), the maximum social welfare function of the village is w(D+f-C-M(n)). If and only if the social welfare (w(D)) that the village committee chooses not to promote agricultural land transfer is lower than the social welfare (w(D+f-C-M(n))w(D+f-C-M(n)) that the village committee promotes agricultural land transfer, then the choice to promote agricultural land transfer is the optimal strategy for the village committee. That is:

D+f(x)−C−M(n)≥D (3)

f(x)≥C+M(n) (4)

The area of land involved in the circulation (xx^R^)x^R^) increases with a decrease in the budget of the village committee (D). Once the village committee chooses to adopt the strategy of promoting the circulation of land (Z = 1) to alleviate financial difficulties, the higher the village-level debt (D ↓↓), and the greater the circulation area (i.e., the optimal area, xx^R^), the financial balance must be maintained. Therefore, when the debt of a village is higher, it has a lower disposable budget. To relieve the debt pressure and change the current situation, the village committee is more likely to promote the circulation of land to relieve financial difficulties, and the higher the debt is, the larger the circulation area that will be promoted.

Based on the above theoretical model analysis, this paper proposes the following hypotheses:

***Hypothesis 1*:** *Village-level debt is one of the reasons for the village committee to intervene in land transfer; and*
***Hypothesis 2*:** *There is a positive correlation between village-level debt and rural land circulation, that is, village-level debt has a significantly positive promoting effect on rural land transfer*.

## 3 Econometric model and variable selection

### 3.1 Econometric model

This study uses panel data, of which the core variables—the village-level debt situation—varies in different areas and is subject to change with time. At the same time, omission variables may occur. To avoid the existence of an area and time effect, with a double fixed effect model was used to empirically analyze the core variables’ effect on the development level of rural land circulation, the control of the village labor force characteristics at the county level at the same time, the land features, the county economy development level, and other control variables, to reduce the core variable coefficient errors. The basic model is as follows.

$$lnY_{it}=\alpha +v.lnL_{it}+\sum \beta .X_{it}+\gamma_{t}+\theta_{i}+\varepsilon_{it}$$ (5)

where Y_it_ represents the transfer area of county i in year t, includes the total transfer area, the area of land flowing into households, the area of land flowing into cooperatives, and the area of land flowing into firms. L_it_ indicates the bundle of village-level debt for county i in year t, subdivided into total debt, operating debt, public welfare debt, village administration, historic debt, and village debt per capita. X_it_ is a set of control variables for county i, including the county GDP, the rural per capita annual income, the rural per capita annual expenditure, the village investment income, the village economic cooperative, the labor force, the number of transfer issues, the completion rate of land transfer, and the cultivated land quantity, while α and vectors v and β represent the coefficients to be estimated. ε_it_ is the random disturbance term, and γ_t_ and θ_i_ represent the year fixed effect and county fixed effect, respectively.

### 3.2 Variable selection

The data in this paper are from two sources: county-level panel data on rural land transfers, and village-level debt from the Zhejiang Provincial Department of Agriculture from 2013 to 2017 and economic data from the Zhejiang Statistical Yearbook, which included total debts, land circulation scale, number of village economic cooperatives, labor force, number of transfer disputes, land approval completion data from Zhejiang Province Agriculture Department of Statistics, background and county data, county cultivated land quantity, county farmers’ per capita disposable income, and per capita consumption expenditure data from the county. The county GDP, amount of cultivated land, per capita disposable income of rural households, and per capita consumption expenditure data are from the Zhejiang County-level Statistical Yearbook from 2013 to 2017.

The definitions and statistical characteristics of the explained variables, core explanatory variables, and control variables are listed in Table 1.

1. Dependent variable. The scale and direction of land transfer, which can be divided into the total area of land transfer, area of land flowing into farmers, area of land flowing into cooperatives, and area of land flowing into firms. There is also a category of circulation subjects, collectively referred to as “others,” which is mainly aimed at circulation objects outside the village that cannot be included in agricultural enterprises. Because its meaning is not clear, it is not included in the regression analysis. Among them, households refer to the households who take part in land transfer and receive a large amount of land from others in the village; cooperatives are professional cooperatives based on the capital in the village and led by the members of the village, while enterprises and other subjects refer to large-scale circulation subjects that contain non-local capital (non-local capital or government-enterprise cooperation projects).
2. Core explanatory variables. When various village debt variables combined with the reasons for the formation of debts, the following three types of debts are obtained after classification and sorting-operating: public welfare debt, village administration debt, and historic debt. Among them, public welfare debts include both material infrastructure debts and social infrastructure debts, while village administration and historic debt include accumulated liabilities of tax borrowing before agricultural tax reform, and loans for the unreasonable expenditure of village organizations. Since the problem of heavy village-level debt has existed for a long time in China [30], the new debt cannot fully reflect the economic problems of villages, so the total amount of debt was chosen in this empirical analysis.
3. Control variables. Based on the existing literature on the influencing factors of farmland transfer, this study selects the quantity of village economic cooperatives and labor force to reflect the characteristics of the rural labor force in villages, and the disputes of land transfer and the completion rate of land rights confirmation are selected to reflect the characteristics of village land. At the same time, the county GDP, amount of arable land, per capita disposable income of farmers, and consumption expenditure are selected as the characteristics of regional economic development.

**Table 1:** Definitions and descriptive statistics of variables.

Variable	Definition/Unit	Obs	Mean	S.D.	Min	Max
Land transfer area variables (dependent variables)						
Total transfer area	hectare	458	7202.796	5425.441	0	22802.6
Land flowing into households	Area of land flowing into households/hectare	458	4489.876	3949.441	0	19533
Land flowing into cooperatives	Area of land flowing into cooperatives/hectare	458	1450.577	1257.543	0	7785.267
Land flowing into firms	Area of land flowing into firms/hectare	458	542.6029	667.529	0	4055.133
Village debt variables (independent variables)						
Total village debt	The total amount of debt borrowed by the village committee of this county/10,000 yuan	458	187001	227001.3	0	1285714
Operating debt	The amount of money borrowed by the village committee of this county because of the business/10,000 yuan	458	18909.64	45980.86	0	385291.7
Public welfare debt	The amount of money borrowed by the county’s village committee for public infrastructure/10,000 yuan	458	8152.228	10646.73	0	80605
Village administration and historic debt	The total amount borrowed by the county’s village committee for administrative expenses and the amount of historical debt/10,000 yuan	458	159948.6	197526.8	0	1141760
Per capita of village debt	This county rural per capita needs to bear the amount of village level debt (total village debt/village population in the county)/yuan per person	456	8417.344	19737.06	152.5254	173011.8
Economic characteristics						
County GDP	GDP in this county/one million yuan	443	557.2189	645.2178	23.5612	3850
Rural Per Capita Annual Income	Rural Per Capita Annual Income in the county/yuan	443	22390.06	5615.48	9013	34316
Rural per capita annual expenditure	Rural per capita annual expenditure in the county/yuan	443	16222.78	3687.865	6135	25421
Village investment income	village investment income in the county/10,000 yuan	458	268356.2	307451.6	0	1828316
Labor characteristics						
Village economic cooperative	The number of village economic cooperative	458	64.93668	149.0346	0	993
Labor force	Number of rural labor force/10,000 persons	458	26.18028	28.38775	0	504.78
Land characteristics						
Transfer issues	Number of land transfer disputes	458	9.670306	27.26084	0	240
Completion rate of land tenture	Number of villages that have completed the right Confirmation/total number of villages (%)	458	23.95354	39.93465	0	100
Cultivated land quantity	Cultivated land quantity in the county/ha	439	22597.06	17992.77	0	115221.3

Note: Economic characteristics, labor characteristics and land characteristics are the control variables. There is another kind of flow subject in the flow, "others", but it is not clear that the object it contains is included, so it is not put into the model for testing.

According to data from the China Rural Development Research Report (2017) released by the Institute of Rural Development of the Chinese Academy of Social Sciences, in 2015, the area transferred to other areas outside the township accounted for 12.72%, which further increased to 16.19% in 2016. This shows that 16% of the transferred land is operated by operators outside the village, and the remaining 84% is cultivated by operators in other villages. According to the calculation, from 2013 to 2017, the average land transferred to other villages in Zhejiang Province accounted for approximately 17.67%, which was close to the national data, indicating that these data are representative to a certain extent. Moreover, part of the control variables of economic development and land characteristics in this study are all from the provincial statistical yearbook, which can reflect the basic characteristics of Zhejiang Province and have practical significance.

## 4 Descriptive analysis

**Fig 1:**
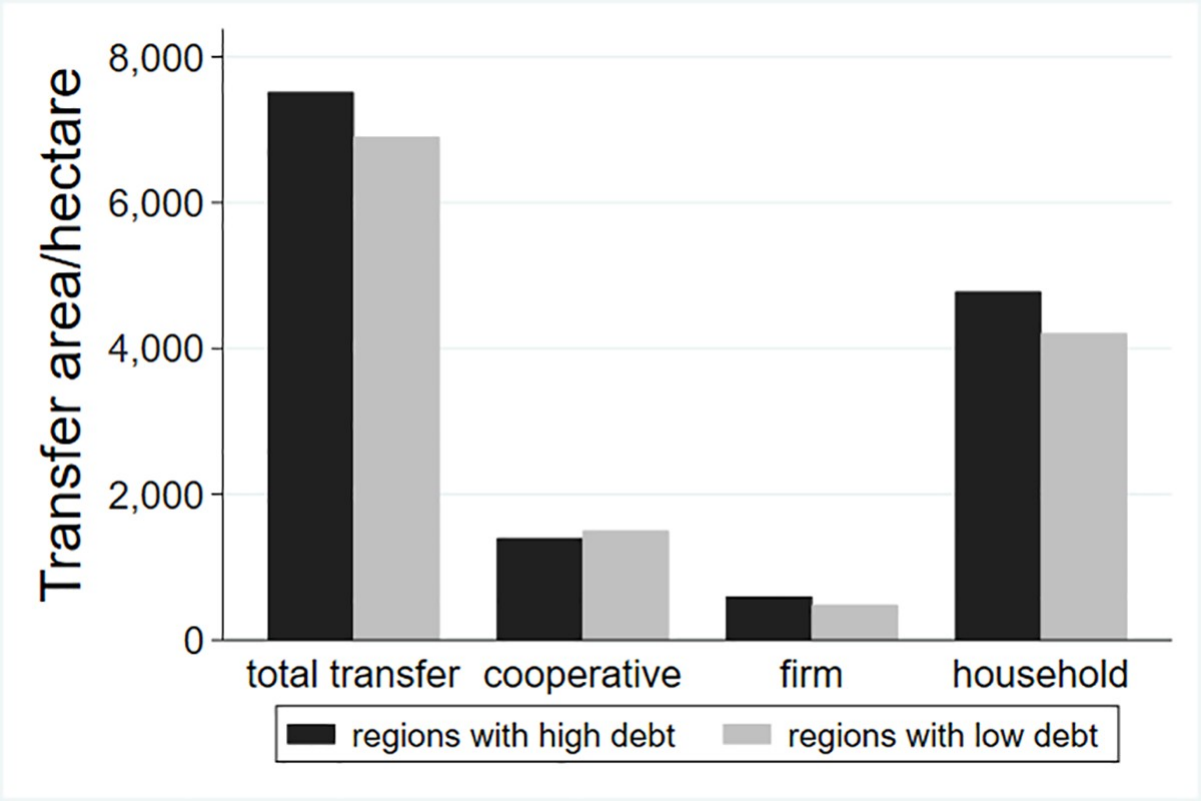
Land transfer scale in the regions with different total debt.

**Fig 2:**
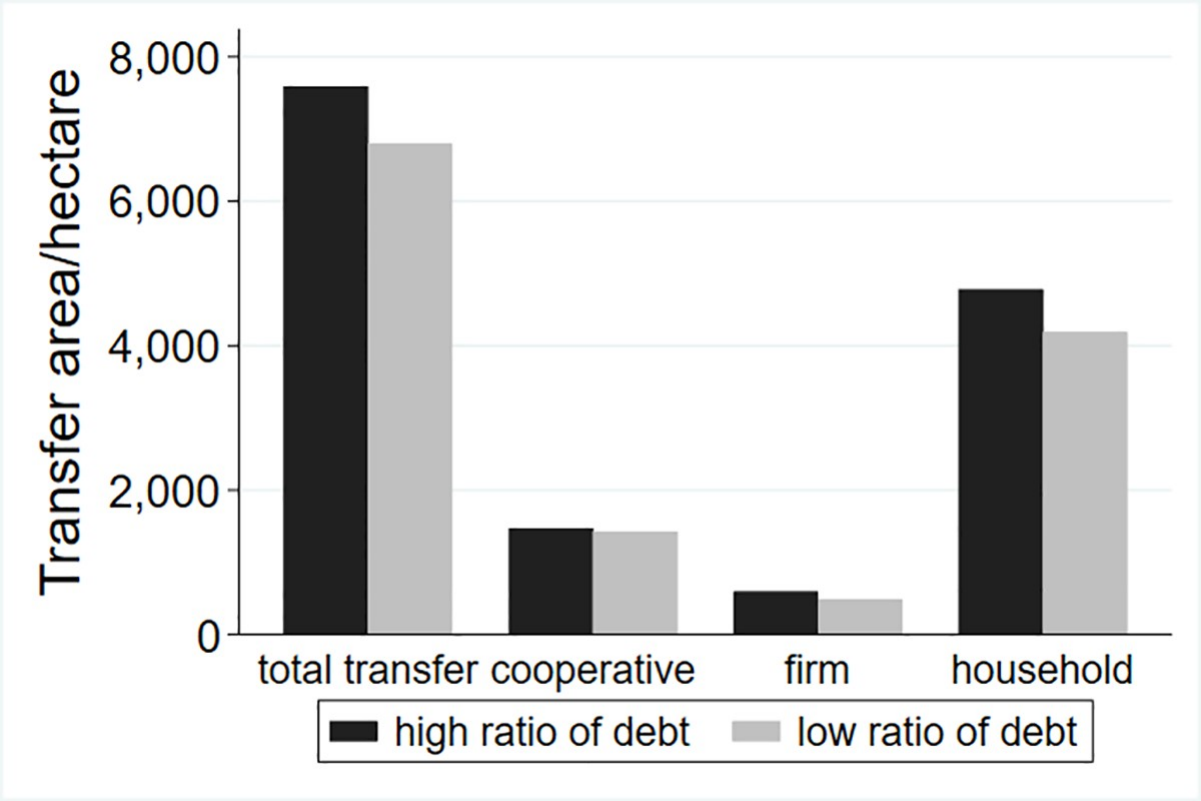
Land transfer scale in the regions with different total per capita debt.

**Fig 3:**
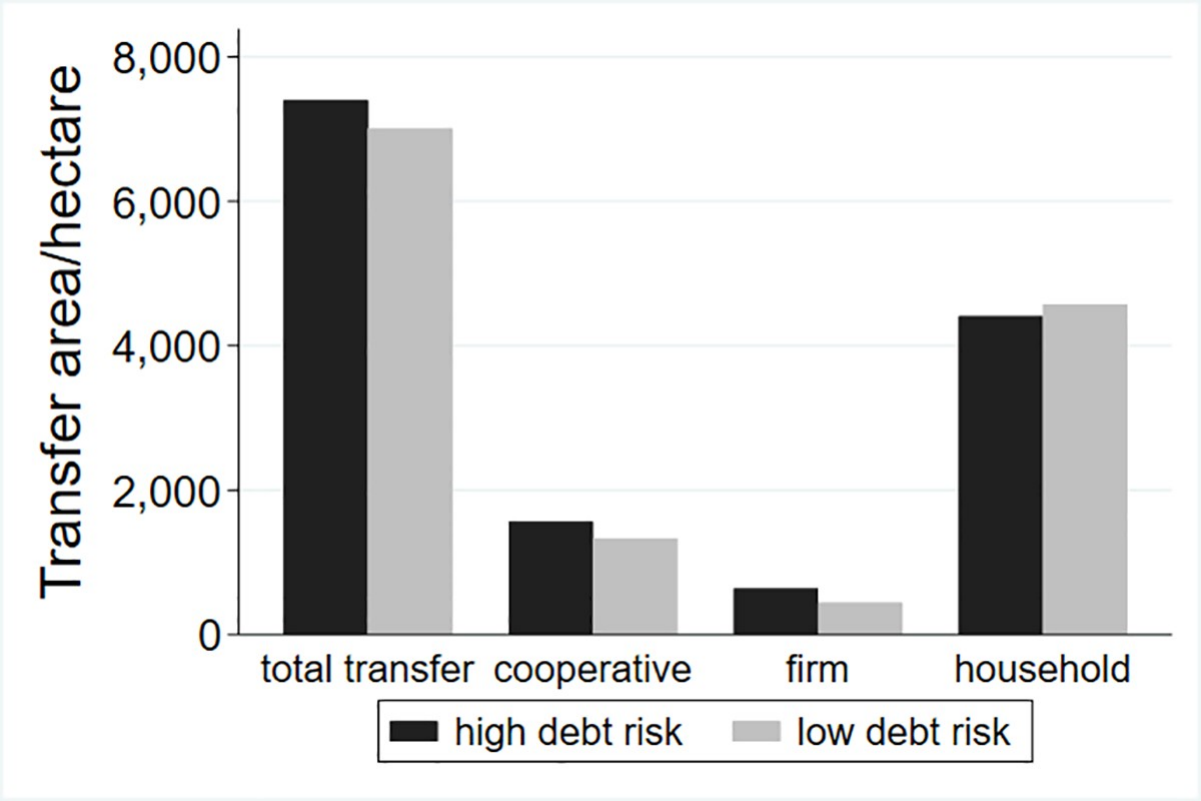
Land transfer scale in the regions with different ratio of debt with per capita income.

This study classifies the debt risk of different villages and examines the differences in land transfer. Du S. (2009) put forward the indicators to measure the scale of rural debt, including debt dependence, debt ratio and debt service ratio, as well as farmers’ per capita debt amount, farmers’ personal debt burden ratio and asset-liability ratio, etc. Two of the indicators were selected in this paper. According to the existing literature, the debt risk of a village cannot be measured through the total debt alone, so this study adds the two indexes of per capita debt and the debt burden rate of farmers. The classification results are shown in Figs 1–3.

As can be seen from Fig 1, the total transfer area of villages with high total debt is significantly higher than that of villages with low total debt, and the land flowing to large agricultural households and enterprises is also higher than that with low total debt. However, the area flowing to the cooperative is contrary, and areas with low per capita debt inflow more land. From the perspective of structure, most of the land flows into households, followed by land flowing into cooperatives, with the least amount of land flowing into firms.

Fig 2 distinguishes the difference in the scale and structure of land circulation between the regions with per capita debt ranking in the top half, and the regions with total debt ranking in the bottom half. As can be seen from Fig 2, the total transfer area of villages with high per capita debt is significantly higher than that of villages with low per capita debt, and the land flowing to households, cooperatives and enterprises is slightly higher than that of villages with low per capita debt. From the perspective of structure, most of the land flows into households, followed by land flowing into cooperatives, with the least amount of land flowing into enterprises.

Fig 3 distinguishes the difference in the scale and structure of land transfer between the regions in the top half of per capita debt-to-income ratio, and the regions in the bottom half of total debt. As we can be seen from the Fig 3, the total circulation area of the villages with high per capita debt-to-income is significantly higher than that of the villages with low per capita debt-to-income, and the land flowing to cooperatives and enterprises is also higher than that of villages with low per capita debt-to-income, while the land flowing to households is lower than that of villages with low per capita debt-to-income. From the perspective of structure, most of the land flows into households, followed by the cooperatives, with the least amount of land flowing into firms, which is consistent with the previous figure.

This reflects that the scale of village-level debt and the per capita debt risk will have an impact on rural land circulation, and this impact is not always positive, but is related to debt risk, village population, and per capita disposable income.

## 5 Result and discussion

### 5.1 Baseline regression

This study uses the county-level panel data of Zhejiang Province from 2013 to 2017 to analyze this problem. First, we check the influence of village debt on the total amount of transfer and structure of transfer in the village, and then analyze the influence of different types of debt on the total circulation level to find the most critical debt type and the actual transferring subject, and the two are cross-tested. It is necessary to judge the reliability of the data and the rationality of the variable design before processing data, that is, to test the reliability and validity of the samples. The reliability test shows that the Cronbach’s coefficient, ɑ, is 0.6168, which is range from 0.6 to 0.8, indicating that internal reliability of data is good; based on the outcome of Table 2,the value of KMO Sampling suitability test for selected explanatory variables is 0.623 (over 0.5), moreover, the Bartlett spherical test mean equals to 978.224, and its adjoint probability is 0.000 (less than 0.01), reaching the extreme significance level and rejecting the zero hypothesis of the Bartlett spherical test,which shows that the explanatory variables selected in the model have good structural validity; besides, the correlation between the variables was determined by the spearman coefficients, most of the systems were significant and below 0.5, and the mean of the variance expansion factor (VIF) was 2.78, well below 10, indicating that there was no multiple collinearity.

**Table 2:** KMO inspection and Bartlett inspection.

Test Category	Parameter	Value of parameter
KMO Sampling suitability test	KMO	0.623
Bartlett spherical inspection	Apcard square	978.224***
Df (Degrees of freedom)	36	
Significance	0.000	

*** p < 0.01, ** p < 0.05, * p < 0.1.

According to the results in Table 3, the total debt level at the village level has a significantly positive impact on the overall circulation scale of the village. Every increase of 10,000 yuan in the total debt level will increase the area participating in the land transfer by 0.008 hectares, indicating that the amount of village debt will significantly promote the land transfer scale of the villages in the county, and the influence of debt on the quantity of land acquired by the three types of transfer subjects is differentiated. Village debt alone has a significant positive effect on the area of the land flowing into firms. When village-level debt increases by 10,000 yuan, the area of land flowing into firms will increase by 0.002 hectares. This shows that when total debt increases, the village is more inclined to transfer land to firms with foreign capital and a higher professional scale, which indicates that under the intervention of the village committee, villages are more willing to choose firms that can produce a certain income and return to be the most suitable transfer subject. In short, the effect of local village debt is not entirely negative for village development. It can promote the scale of local land transfer to some extent, adjust the transfer structure, and push land concentrated in firms, which is also consistent with the obvious characteristics of profit-seeking for cooperatives in Zhejiang Province [30]. Besides, the number of economic cooperatives, the size of the labor force and the land disputes in the village have a positive and significant impact on the transfer of land. That is, more people are engaged in agricultural farming with more labors in the village. Thus, the amount of land transfer in the land circulation market will increase, and the total scale of land transfer will increase accordingly; the number of land transfer disputes reflects the transaction situation of the land market to some extent, with more disputes indicating that more farmers will participate in land transfer, which means that more land will be transferred, the more farmers that farmers can receive transfer services, and the more they can get timely information to reduce the transaction costs in the transfer with more economic cooperatives in the village.

**Table 3:** The estimation results of the relationship between village-level debt and rural land transfer.

	Total transfer area	Land flowing into households	Land flowing into cooperatives	Land flowing into firms	Total transfer area	Total transfer area
	(1)	(2)	(3)	(4)	(5)	(6)
Total village debt	0.00863***	0.00378	-0.000644	0.00184***	0.00433	0.0126***
	(0.00308)	(0.00249)	(0.00112)	(0.000681)	(0.0112)	(0.00400)
County GDP (ln)	90.22	166.2	23.64	-66.34	2,158	-199.3
	(478.2)	(385.6)	(174.0)	(105.6)	(2,056)	(734.3)
Rural per capita annual income (ln)	688.8	1,142	212.5	-3.411	2,069	-2,001
	(1,615)	(1,302)	(587.5)	(356.7)	(1,916)	(3,210)
Rural per capita annual expenditure (ln)	675.4	215.1	-19.43	-160.1	-857.6	211.5
	(1,821)	(1,468)	(662.4)	(402.1)	(2,247)	(4,807)
Cultivated land quantity	96.91	159.9	-148.9	9.123	-264.0	337.3
	(502.8)	(405.3)	(182.9)	(111.0)	(807.3)	(673.6)
Village economic cooperative	2.533**	1.794**	0.541	0.0405	1.887	1.865
	(0.985)	(0.794)	(0.358)	(0.218)	(1.345)	(1.597)
Labor force	258.5***	178.4***	35.63***	13.08***	224.7***	272.0***
	(10.16)	(8.192)	(3.696)	(2.244)	(17.52)	(13.77)
Land transfer disputes	18.41***	9.676**	9.349***	-0.838	13.72**	23.32***
	(5.031)	(4.056)	(1.830)	(1.111)	(6.623)	(8.086)
Completion rate of land tenture	2.521	0.886	-0.461	0.410	1.586	3.068
	(6.791)	(5.475)	(2.470)	(1.500)	(8.758)	(10.31)
Constant	-15,305	-16,162	126.6	1,828	-19,573	12,022
	(18,643)	(15,030)	(6,781)	(4,117)	(26,970)	(50,306)
County FE	√	√	√	√	√	√
Year FE	√	√	√	√	√	√
Obs	417	417	417	417	211	206
R^2^	0.696	0.625	0.328	0.120	0.577	0.769

Note: a. Model (5) and Model (6) are classified according to the debt ratio (county and village debt/county GDP), Model (5) refers to the counties in the lower half of the village debt rate, that is, the regions with low debt rate (less than 2.9%); Model (6) refers to the counties in the top half of the village debt rate, that is, the regions with high debt rate (more than 2.9%). b. Robust standard errors are in parentheses.

*** p < 0.01

** p < 0.05

* p < 0.1.

According to the existing literature, the positive effect of village debt will be limited by the actual amount of debt, and it is doubtful whether debt has been driving land transfer. Therefore, this study divides counties into two categories according to the debt ratio (total debt of lower villages at the county level/county GDP): counties with debt ratios in the top and bottom halves, respectively. The results are shown in Columns 5 and 6 in Table 3. When the debt ratio is lower than 2.9%, the promotion effect of village debt on transfer is not significant, while the opposite is true, indicating that there is a threshold value for the promotion effect of debt. Therefore, this study takes the debt ratio as the classification standard to explore the driving effect of county and village debt on land circulation under different debt ratios. The results are shown in Table 4. According to the regression results below, the driving effect of village debt on land circulation is related to the debt ratio, namely the risk of village debt within the county, and there is an upper limit and a lower limit on the amount of debt. It can be preliminarily estimated that when the debt ratio of the village is between 4.65% and 7.9%, debt plays a significant role in promoting land transfer. Hypothesis 2 is proved.

**Table 4:** The estimation results of village-level debt and rural land transfer in different debt_tatio.

	Total transfer area						
	(1)	(2)	(3)	(4)	(5)	(6)	(7)
	(debt_ratio >4.63%)	(debt_ratio >4.65%)	(debt_ratio >4.7%)	(debt_ratio >6%)	(debt_ratio >7%)	(debt_ratio >7.9%)	(debt_ratio >8%)
Total village debt	0.00815	0.00919*	0.0101*	0.0184**	0.0333**	0.0696*	0.0319
	(0.00501)	(0.00505)	(0.00513)	(0.00709)	(0.0147)	(0.0368)	(0.0466)
Covariate	√	√	√	√	√	√	√
County FE	√	√	√	√	√	√	√
Year FE	√	√	√	√	√	√	√
Obs	119	118	113	71	49	38	37
R^2^	0.796	0.799	0.805	0.805	0.871	0.894	0.908

Note: ^a.^ Covariates contain County GDP (ln), rural per capita annual income (ln), rural per capita annual expenditure (ln), the number of village economic cooperative, the number of village labor force, Land transfer disputes in the county and completion rate of land tenture; ^b.^ The *t*-statistics are shown in parentheses. Robust standard errors are in parentheses. *** *p* < 0.01 ** *p* < 0.05 * *p* < 0.1.;^c.^ Due to the limitation of space, the estimation results of control variables are omitted. d. debt_ratio means total village-level debt of the county divided by county GDP.

The above study on the total amount of village debt found that it could promote the circulation of land to some extent, but further verification is needed to determine which type of debt plays a key role. Therefore, this study further carried out the regression of the fixed effect model between different types of debt and the scale of total circulation of farmland, and obtained the estimated results shown in Table 5. As can be seen from Column 3 in Table 5, village administration and historic debt can significantly positively affect the circulation of land. Among them, the total scale of land circulation will increase by 0.008 hectares for every 10,000 yuan increase in debt. Column 4 checks the relationship between village administration and historic debt and land flowing to firms, and the results are significant, which indicates that village administration and historic debt are the liabilities that promote the circulation of agricultural land to promote the large-scale operation of land.

**Table 5:** The estimation results of the relationship between various village debt and total land transfer.

	Total transfer area			Land flowing into firms
	(1)	(2)	(3)	(4)
Operating debt	0.00301			
	(0.00664)			
Public welfare debt		0.0379		
		(0.0290)		
Village administration and historic debt			0.00806**	0.00157**
			(0.00320)	(0.000707)
Covariate	√	√	√	√
County FE	√	√	√	√
Year FE	√	√	√	√
Obs	417	417	417	417
R^2^	0.689	0.690	0.694	0.114

Note: ^a.^ Covariates contain County GDP (ln), rural per capita annual income (ln), rural per capita annual expenditure (ln), the number of village economic cooperative, the number of village labor force, Land transfer disputes in the county and completion rate of land tenture; ^b.^ Robust standard errors are in parentheses. *** *p* < 0.01

** *p* < 0.05, * *p* < 0.1.;^c.^ Due to the limitation of space, the estimation results of control variables are omitted.

### 5.2 Trade-off

One of the reasons for village debt to promote the transfer of land in the village is the village committee’s pursuit of its own interests. Land transfer in China requires the approval of the village committee [58], and the village committee has the motivation to regulate the transfer of agricultural land. First, according to the Organic Law of the Village Committees of the People’s Republic of China, village committees provide and maintain the rules for the transfer of the right to use rural land on behalf of the rural grassroots political power [59]; second, members of the village committee, the so-called village cadres, supervise the transfer of land out of their own interests, as regulating the transfer of agricultural land provides them with rent-seeking opportunities [60]; in other words, the village committee has endogenous power and the right to control the circulation of land.

The village committee serves as a middleman, and provides services for farmers with the land circulation of agricultural firms; in the meantime, reducing the transaction costs will help the farmers’ land circulation, with the village collective economic organizations getting most of the villagers to sign a contract of land circulation. Some of the village collective economic organizations or the village committee cadres sign the contract as a contacting third party, while in other places, the village committee directly restrains farmers’ contracted land to seize the opportunity of earnings, so as to become a part of the village’s investment gains [61]. Shi et al. (2018) conducted a survey of 58 villages from four counties in Jiangsu and Jiangxi Provinces, including 1,800 households. It was found that nearly 48.2% of the farmers are interventions for village land transfer, which includes two modes: (a) transfers of land that require the consent of the village committee; and (b) transfers of land where the usage or assignor is restricted, and it is decided by the village committee. This study found that the active restriction and intervention of village committees in land transfers would reduce farmers’ willingness to participate in land transfers [62]. D.F. Wang et al. (2011) and Sun et al. (2017) conducted a survey in a rural town in southern Anhui Province. They found that the strong intervention of the government cut off the virtuous circle of spontaneous transfer of agricultural land, but formed the mechanism of “village committees acting as executors and county and township governments behind the promotion of transfer,” which indicated that “village committees intervening in land transfer” exist objectively in China [63,64].

Based on the research findings, this study considers village investment income as the dependent variable, and the total debt of the village and the transaction debt of the village organization as the independent variable. After controlling for the variables of the village population, total income, labor force, and arable land area, the analysis results are presented in Table 6. There is a significant positive correlation between village debt and village investment income, which indicates that the village debt will indeed become a big driving force for the village committee to seek investment from outside. It also indicates that the intervention of the village committee in the land market is not without seeking return, which is supported by Hypothesis 1.

**Table 6:** The estimation results of the relationship between village-level debt and investment revenue.

	Village investment income	
	(1)	(2)
Total debt	0.288**	
	(0.076)	
Village administration and historic debt		0.360**
		(0.077)
Covariate	√	√
County FE	√	√
Year FE	√	√
Obs	417	417
R^2^	0.428	0.440

Note: ^a.^ Covariates contain County GDP (ln), rural per capita annual income (ln), rural per capita annual expenditure (ln), the number of village economic cooperative, the number of village labor force, Land transfer disputes in the county and completion rate of land tenture; ^b.^ Robust standard errors are in parentheses. *** *p* < 0.01

** *p* < 0.05, * *p* < 0.1.;^c.^ Due to the limitation of space, the estimation results of control variables are omitted.

### 5.3 Robustness check

In addition to the total debt of the village to measure the debt pressure of the village, this study also uses the per capita debt of the village to reflect the debt repayment risk of the village and carry out a robustness check.

The regression results are shown in Table 7. As we can see from Columns 1 to 4, the per capita assumption of village-level debt will significantly increase the total scale of land circulation. When the per capita assumption of village debt increases by 10,000 yuan, the total area of land transfer will increase by 0.125 hectares. In addition, the area of land flowing into firms also increased significantly. When the per capita village debt increased by 10,000 yuan, the area of land flowing into firms increased by 0.03 hectares. Although the area flowing to households and cooperatives was positive, it was not significant, which was consistent with the previous results, indicating that the conclusion of this study was robust.

**Table 7:** Robust check of influence of village debt on farmland transfer.

	Total transfer area	Land flowing into households	Land flowing into cooperatives	Land flowing into firms	Village investment income
	(1)	(2)	(3)	(4)	(5)
Per capita of village debt	0.125**	0.0194	0.00739	0.0294**	3.664***
	(0.0530)	(0.0427)	(0.0192)	(0.0117)	(1.303)
Covariate	√	√	√	√	√
County FE	√	√	√	√	√
Year FE	√	√	√	√	√
Obs	417	417	417	417	417
R^2^	0.694	0.624	0.328	0.117	0.417

Note: ^a.^ Covariates contain County GDP (ln), rural per capita annual income (ln), rural per capita annual expenditure (ln), the number of village economic cooperative, the number of village labor force, Land transfer disputes in the county, completion rate of land tenture and whether the county experience agricultural tax reform or not (dummy variable); ^b.^ Robust standard errors are in parentheses.

*** *p* < 0.01

** *p* < 0.05, * *p* < 0.1.; ^c.^ Due to the limitation of space, the estimation results of control variables are omitted.

In other words, when the per capita debt repayment pressure increases, villages in the county are more inclined to pour land into entities such as enterprises, indicating that under the intervention of the village committee, villages are more inclined to choose enterprises that can obtain certain income returns. At the same time, this article also reveals the village debt per capita conduction mechanism of the inspection. When investment income per capita of village-level debt and village has a significant positive correlation, but the coefficient is significantly higher than other means when the village debt risk increases, this study finds that the average per capita debt service pressure increases. It not only promotes the village committee to seek outward investment, but also increases the strength and intensity of its seeking, which shows that besides total debt, the share of the debt level is another level of pressure on the village committee to pursue their own interests.

### 5.4 Heterogeneity analysis

Heterogeneity, which is used to describe the effect of variation in a series of studies, also shows that there are differences between studies except for a foreseeable chance, which can help us to see the different reflections of individuals or regions with different characteristics under the influence of a certain factor; the influence of village-level debt on land circulation may also be heterogeneous. There are two reasons for this. First, Zhejiang Province is in the southeast coastal area of China, and the terrain slopes from southwest to northeast. The terrain is complex, with more mountains and hills, and fewer plains, and the amount of cultivated land resources varies in different regions. Therefore, owing to different resource endowments, there will be significant differences in the scale of land transfer between regions [65]. Second, the proportion of the primary industry in GDP reflects the position of agriculture in regional economic growth, which affects the area of land transfer in the region. Therefore, regions that attach great importance to agriculture will have different promoting effects on land transfer [66].

Table 8 tests the potential of village debt on regional land transfer under different natural economic characteristics. According to the landform, the county is divided into mountains, hilly areas, and plain paddy field areas. Counties are divided into coastal areas and non-coastal areas according to whether it is adjacent to the sea. Finally, depending on the proportion of agricultural GDP in each county, two samples were selected for heterogeneity analysis after classification, which were the counties in the top third of agricultural GDP and the counties in the bottom third of agricultural GDP. The regression results show that village debt has a significant promoting effect on land transfer between plain areas and non-coastal areas, and the possible reasons are as follows: Firstly, the cultivated land resources in the plain area are richer than those in other areas, so the land scale base that can participate in the transfer is larger; Secondly, the farming conditions in non-coastal areas are more suitable than those in coastal areas, and the non-agricultural industries in coastal areas are more developed, which can attract one of the largest rural labor forces in the whole province, including non-coastal areas. Therefore, the amount of land that can be transferred in non-coastal areas increases. In addition, in regions with a high proportion of agricultural GDP, village debt also plays a significant role in promoting land circulation. The possible reason is that regions with developed agriculture are highly dependent on agriculture and more dependent on the improvement of production efficiency brought by large-scale land management. Therefore, village committees will pay more attention to and promote land circulation.

**Table 8:** Heterogeneity analysis of influence of village debt and land transfer.

	Hilly and mountainous regions	Plains	Coastal areas	Non-coastal area	Counties with agricultural GDP rank on the top 1/3	Counties with agricultural GDP rank on the last 1/3
	(1)	(2)	(3)	(4)	(5)	(6)
Total debt	0.00397	0.0156***	0.00532	0.00953*	0.0314**	0.00482
	(0.00392)	(0.00556)	(0.00445)	(0.00536)	(0.0122)	(0.00384)
Covariate	√	√	√	√	√	√
County FE	√	√	√	√	√	√
Year FE	√	√	√	√	√	√
Obs	310	107	170	247	131	132
R^2^	0.672	0.813	0.766	0.645	0.735	0.818

Note: ^a.^ Covariates contain County GDP (ln), rural per capita annual income (ln), rural per capita annual expenditure (ln), the number of village economic cooperative, the number of village labor force, Land transfer disputes in the county and completion rate of land tenture; ^b.^ Robust standard errors are in parentheses.

*** *p* < 0.01

** *p* < 0.05

* *p* < 0.1.;^c.^ Due to the limitation of space, the estimation results of control variables are omitted.

## 6 Conclusion

Promoting rural land scale management has always been an important goal of land reform. The paper takes the village finance as the starting point to analyze the impact of rural debt on the village land transfer. It found that the scale of land transfer becomes larger when the village-level debt is higher, and there are significant differences between the land area flowing to different subjects, and the land area flowing to agricultural enterprises increases significantly. Actually, what really boosts the farmland scale operation is village administration and historic debt. However, there is a threshold for the debt driving action, and the impact is significant when its debt rate is at 4.7% ~ 7.9%. The village committee also participated in the land transfer as an intermediary, and complied with the policy and obtained investment income, to achieve a win—win situation. The paper provides empirical evidence for the following points of view: like the labor market studied by new economic sociologists, China’s rural contracted land rental market has a strong social embedment. Specifically, it is not only embedded in the “acquaintance society” formed by rural villagers, but is also embedded in the governance of the social and economic life of the community. As the grassroots administrator of “acquaintance society,” the village committee not only participates in the circulation of rural land in many roles, but the way and degree of involvement are also affected by the economic situation of the village collective, especially the debt situation. This shows that, as a kind of agricultural land transfer activity that has a profound impact on local lives, it is not only restricted by the market mechanism, but also restricted by the community mechanism. The two mechanisms are embedded together, and it is difficult to form a situation that completely replaces each other. This pattern of mutual embedding of market governance and community governance will not change substantially with the deepening affirmation of property rights and the reduction of transaction costs, as the popular propositions of property right economics and transaction cost economics have concluded. At the same time, both market governance and community governance are embedded in administrative governance, and at the rural grassroots level, the leading role of administrative governance is naturally the village committee.

There are two situations in which village committees participate in land transfer as participants: firstly, village committee provides information service and transfer platform for farmers which reduces the transaction cost of land transfer to promote the standardized and orderly transfer of land among farmers as an assistant; secondly, the village committee transfers the collectively-owned land to other renters-local households or agricultural enterprises-and converts the rent into its own investment income. However, there are differences in the quantity and transfer of land belonging to village collective among villages. Some villages have a large amount of land belonging to village collective, which is transferred to households as well as enterprises. Some villages have a small number of land, so the tenants will be subject to the actual economic and social needs. Based on the outcome of Table 3, when the economic pressure of the village increases, that is, the debt of the village increases, land flowing to agricultural enterprises will increase significantly while land flowing to farmers will not be significantly affected that means village committee tends to choose enterprises as the transfer object of mobile land in order to obtain rent to reduce the economic pressure when the economic pressure of the village becomes higher. Land transfer data in this paper did not distinguish between the land sources participating in the land transfer (i. e., farmers ’ contracted land or land belonging to village collectives), so this time there is no empirical analysis for different land sources.

However, the theoretical contribution of this study is undoubtedly marginal, partial, and tentative. The data used in this study lack information on agricultural land outflow, and aggregate data at the county level cannot reflect the differences among villages. Therefore, the analysis of village-level debt in rural land circulation is still incomplete. Future research can focus on the connotation of community embeddedness of rural land transfers, including the socioeconomic development status of rural communities, the social and economic structure of communities, the structure of grassroots social governance organizations, and even the mode and operation of community embeddedness. Finally, as an organization of rural autonomy in China, the power of village committees to intervene in land transfer comes from two aspects: the power of political power and the personal prestige of village cadres. It is also important to distinguish the influences of the two.

In short, land transfer and land market behavior in a wider context are a rich source of emerging research on community governance, as well as of economic sociology and political economy. It remains to be exploited comprehensively, systematically, and deeply by social science researchers using various qualitative and quantitative methods.

## Supporting information

S1 Dataset (DTA)
